# Dynamic Docking: A Paradigm Shift in Computational Drug Discovery

**DOI:** 10.3390/molecules22112029

**Published:** 2017-11-22

**Authors:** Dario Gioia, Martina Bertazzo, Maurizio Recanatini, Matteo Masetti, Andrea Cavalli

**Affiliations:** 1 Department of Pharmacy and Biotechnology, Alma Mater Studiorum—Universita’ di Bologna, via Belmeloro 6, I-40126 Bologna, Italy; dario.gioia2@unibo.it (D.G.); martina.bertazzo2@unibo.it (M.B.); maurizio.recanatini@unibo.it (M.R.); 2 Computational Sciences, Istituto Italiano di Tecnologia, via Morego 30, 16163 Genova, Italy

**Keywords:** protein-ligand binding, molecular dynamics, enhanced sampling, binding kinetics, drug discovery

## Abstract

Molecular docking is the methodology of choice for studying in silico protein-ligand binding and for prioritizing compounds to discover new lead candidates. Traditional docking simulations suffer from major limitations, mostly related to the static or semi-flexible treatment of ligands and targets. They also neglect solvation and entropic effects, which strongly limits their predictive power. During the last decade, methods based on full atomistic molecular dynamics (MD) have emerged as a valid alternative for simulating macromolecular complexes. In principle, compared to traditional docking, MD allows the full exploration of drug-target recognition and binding from both the mechanistic and energetic points of view (dynamic docking). Binding and unbinding kinetic constants can also be determined. While dynamic docking is still too computationally expensive to be routinely used in fast-paced drug discovery programs, the advent of faster computing architectures and advanced simulation methodologies are changing this scenario. It is feasible that dynamic docking will replace static docking approaches in the near future, leading to a major paradigm shift in in silico drug discovery. Against this background, we review the key achievements that have paved the way for this progress.

## 1. Introduction

Nowadays, molecular docking programs are extensively and routinely used in computer-aided drug discovery, mostly in the framework of virtual-library screening (VS) campaigns [1,2]. This is the first critical step in structure-based drug discovery (SBDD), where the new drug is identified as the ligand that fits best into the binding pocket of the protein target [2]. In the past two decades, researchers have produced a wealth of structural data, and constantly improved the protocols for molecular calculations, allowing for a rapid screening of libraries containing hundreds of thousands of compounds. However, this computational speed comes at the cost of accuracy, especially when target rearrangements are required upon ligand binding [3,4]. Indeed, SBDD still suffers from fundamental issues and limitations [3]. First, and foremost, flexibility is a crucial aspect for correctly estimating the binding geometries (i.e., binding modes, or, in the docking jargon, simply “poses”) [4]. Furthermore, docking algorithms lack explicit water treatments, which are crucial for reproducing specific drug-target complexes. Moreover, the underlying approximations do not allow a reliable estimation of key thermodynamic observables, such as the binding free energy [3]. Finally, these approaches provide only a static picture of the binding process, which means that they cannot estimate kinetic quantities. Notably, binding kinetics is becoming increasingly important for drug discovery and development because it has emerged as a more reliable predictor of in vivo drug efficacy than affinity [5].

All the above mentioned limitations can be addressed by molecular dynamics (MD) simulations and related methods [3]. Thanks to the rapid development of faster architectures (mainly Graphics Processing Unit (GPU)-based clusters) and better algorithms for high-level computations, classical MD simulations nowadays allow the implementation of SBDD strategies that account for the structural flexibility of drug-target systems at a fully atomistic description [3]. Additionally, provided that simulations are long enough to cover the entire drug-binding process (i.e., from the drug fully solvated in water to the drug-target bound state), the thermodynamics and kinetics become straightforward products of MD approaches [3,6]. In principle, simulations lasting up to a few milliseconds are now possible [7]. However, several different trajectories are commonly required to obtain adequate statistics and to exhaustively sample the configurational space. This makes the whole process computationally demanding, even when studying just a single lead compound. As such, “brute force” plain MD simulations do not provide the efficiency that is necessary if a computational tool is to be routinely used to identify drug candidates. This is particularly true when dealing with series of compounds in the hit-to-lead or lead optimization steps of the drug discovery process. Several research groups have addressed these limitations using enhanced sampling methods [8]. This class of MD-based simulative approaches allows one to accelerate the observation of events, such as drug-target (un)binding, by biasing the forces or altering the potential energy function. Given the availability of these theoretical methods and ever-increasing computational power, MD simulations are well-positioned to replace the old-fashioned view of static molecular docking with a novel concept that we call “dynamic docking”.

Herein, we begin by summarizing the theory of traditional molecular docking, highlighting the advantages that have made it so popular over the years, and its major limitations. We then discuss some of the many attempts to introduce MD concepts into docking frameworks. We move on to the main concepts of solvent mapping (or co-solvent) simulations, which we consider to be the turning point toward a fully dynamic prediction of protein-ligand interactions. Finally, we review the most effective and promising methods for dynamic docking. We emphasize the benefits of a dynamic description compared to the static view of binding provided by conventional docking methods.

## 2. Benefits and Limitations of Static Molecular Docking

Molecular docking is a well-established computational approach pertaining to SBDD [9]. It aims to predict the 3D geometry of protein-ligand bound complexes. Kuntz and co-workers [10] reported the first attempts at molecular docking in the early 1980s. Since then, the methodology has continued to evolve. In contrast to the rigid body association exercises of the earliest implementations, full flexibility for the ligand is nowadays allowed. Most recently, researchers have begun to incorporate partial flexibility for the receptor too [11,12]. Another key feature is the exploitation of purposely developed functions that rank the molecular docking solutions according to their binding propensity, thus mimicking estimators of binding free energy. A typical docking algorithm is conveniently split into two distinct steps, the posing phase and the scoring phase. Each phase targets a specific problem [13]. Table 1 provides a short list of currently available docking software.

### 2.1. Posing

During the posing phase, a searching algorithm, which can be either stochastic or systematic (see Table 1), generates a set of configurations of the ligand at the receptor’s binding site. These include both roto-translational and internal (usually conformational) degrees of freedom of the ligand. For practical reasons, mostly due to limited computational resources, the early docking implementations commonly dealt with a single rigid conformation of the target. However, it is inadvisable to neglect target flexibility in docking or VS. This is because proteins are intrinsically dynamic and, most often, there is a mutual adaption upon binding in order to maximize favourable contacts [14].

This picture of binding corresponds to the popular induced fit model proposed by Koshland in 1958 [23]. According to a more recent interpretation of binding, proteins naturally exist in a conformational ensemble. Upon binding, one such conformation is thermodynamically “selected”, resulting in a change in population with respect to the equilibrium in the apo form (conformational selection or population shift model) [24,25]. A greater understanding of protein-ligand recognition mechanisms has prompted a gradual introduction of the receptor’s degrees of freedom in standard docking procedures. In principle, the degrees of freedom of the receptor and ligand should be sampled simultaneously, but the computational complexity of the problem prevents such a naïve approach. Instead, the pragmatic strategies mirror the previously mentioned mechanisms of recognition [14]. They can be classified as either single-structure methods or ensemble methods. In single-structure methods, the binding pocket is perturbed by on-the-fly local changes, mimicking the induced fit model. In ensemble methods, an ensemble of previously generated conformations (either theoretically or experimentally) is exploited in a serial docking exercise in the spirit of the population shift model. Ensemble methods, in particular, are gaining in popularity for SBDD (see below). Despite this, the information provided by multiple protein conformations is not always straightforward to manage, and it must be properly handled to be effectively exploited [26,27,28]. Moreover, all these strategies offer very elaborate and sometimes very involved solutions to a problem that could be naturally addressed with MD simulations [3,6].

### 2.2. Scoring

In the second stage of a docking protocol, the binding modes retrieved during sampling must be evaluated. The noncovalent association between a protein (*P*) and a ligand (*L*) to form the complex (*PL*):

(1) P+L⇆PL

is described by the equilibrium (or association) constant *K*_a_:

(2) $$K_{a}=\frac{\left[PL\right]}{\left[P\right]\left[L\right]}=\frac{1}{K_{d}}$$

The association constant, or its reciprocal dissociation constant, *K*_d_, is related to the standard free energy of binding as follows:

(3) $$\Delta G_{b}^{{}^{\circ}}=-k_{B}T\ln\left(K_{a}\;C^{\circ }\right)=k_{B}T\ln\left(\frac{K_{d}}{C^{\circ }}\right)$$

where *k*_B_ is the Boltzmann constant and *C*° is the standard concentration. Calculating free energy differences is not a trivial task. Together with other entropy-related quantities, free energy is the thermodynamic observable whose estimation suffers the most from sampling limitations and underlying approximations. From a statistical mechanics standpoint, Equation (3) translates to [29]:

(4) $$\Delta G_{b}^{{}^{\circ}}=-k_{B}T\ln\left(\frac{Z_{PL}}{Z_{P}Z_{L}}C^{\circ }\right)$$

where *Z_PL_*, *Z_P_* and *Z_L_* are the configurational integrals of the bound complex, the protein, and the ligand, respectively. For each *i*-th species, the configurational integral *Z_i_* can be further expressed as [29]:

(5) $$Z_{i}=\int^{\text{​}}e^{-\left(U\left(x_{i}\right)+W\left(x_{i}\right)\right)/k_{B}T}dx_{i}$$

where *U*(**x***_i_*) and *W*(**x***_i_*) are the potential energy and the solvation free energy of the *i*-th component in the configuration **x**. It is clear that, in order to assess the configurational integral, a proper description of the solvent environment and exhaustive sampling are required. In other words, both (de)solvation effects and entropy changes upon binding must be taken into account to obtain reliable free-energy estimates. These aspects of protein-ligand binding, however, require computationally expensive procedures that are usually precluded by molecular docking calculations. Scoring functions (Table 1) attempt to address this problem, either by introducing several approximations (mainly in the solvent treatment and by neglecting entropic contributions, force-field based scoring functions) or by adopting convenient phenomenological descriptions (empirical and knowledge-based scoring functions) [30]. We note that scoring functions should not only identify the fittest binding mode from all the poses generated by the posing algorithm, but, in a VS campaign, they should also discern binders from non-binders (ranking problem) [31]. It is nowadays widely accepted that docking methods can identify the experimental binding mode, at least when protein flexibility does not play a relevant role. However, the ranking problem remains an open issue. Again, a dynamic docking framework would ensure the proper theoretical foundation required to obtain reliable energy estimates [3,6].

## 3. Plugging MD into Static Modeling Frameworks

As is often the case with paradigm shifts in computational approaches, the route leading from static to dynamic docking was not straightforward. Awareness of the need for a dynamic description of protein-ligand interactions has grown gradually, rather than in an all-or-nothing fashion. Several workarounds have therefore been developed, mainly based on combined docking MD strategies. In parallel, however, boosted by the increase in computational power, researchers began applying MD approaches to a different, albeit related, drug discovery problem. These are the so-called solvent mapping or co-solvent MD simulations, which can nowadays be used to identify binding sites and hot-spots on protein surfaces at a fully dynamic level.

### 3.1. Combining Docking and Molecular Dynamics Simulations

**Figure 1:**
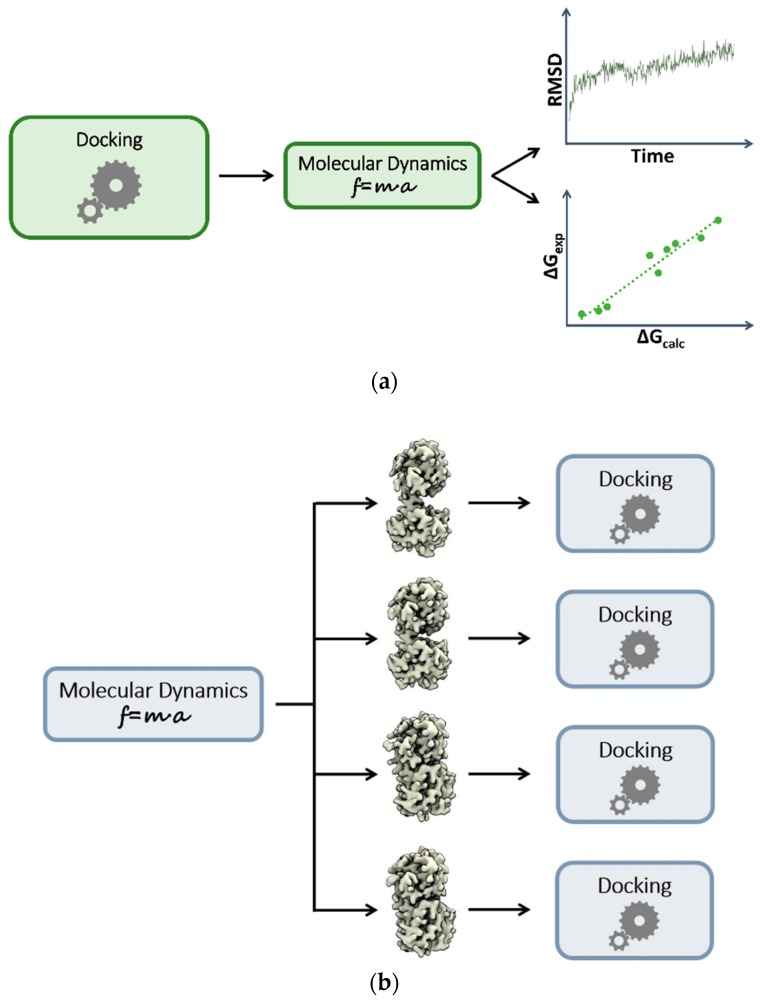
Sequential combination of docking and MD simulations. (**a**) MD employed for rescoring or refining docking poses; (**b**) MD employed for conformational ensemble generation. Docking is then performed against multiple rigid receptor conformations.

The static picture provided by conventional docking methods still prevails. However, several attempts to implement dynamic aspects of binding have been reported. These efforts are based on a sequential combination of docking and MD. Depending on the order in which they take place, different issues can, in principle, be addressed. For example, MD simulations that start from docking outcomes are typically used to validate or refine results with higher-level theories or sampling approaches (Figure 1a). In particular, two kinds of refinement can be applied. From a structural standpoint, MD simulations can reveal unstable binding modes, helping to filter out physically unreliable docking solutions, or even helping to identify new ones [32,33,34]. Here, the advantage of MD is that flexibility is fully accounted for and induced fit effects are naturally included, albeit only afterwards. From a different perspective, post-processing through MD can also be used to refine the energetics estimated by scoring functions (re-scoring). Re-scoring approaches use a wide range of theoretical methods [35], but they all differ from simple scoring functions in that the energies are computed as ensemble averages, approaching a more rigorous description of binding. Re-scoring schemes range from partially empirical methods such as Linear Interaction Energy (LIE [36]) through to authentic free-energy approaches such as Free-Energy Perturbation (FEP [37]) or Thermodynamic Integration (TI [38]). Between these extremes lies probably the most popular solution, the Molecular Mechanics-Poisson-Boltzmann Surface Area and Molecular Mechanics-Generalized Born Surface Area (MM-PB/GB SA) re-scoring method, which provides a good balance between reasonable accuracy and computational costs [36]. More recently, researchers have begun applying enhanced sampling methods in the spirit of re-scoring or re-ranking approaches [39,40,41,42].

Another widely adopted solution is to perform MD prior to docking in order to generate an ensemble of protein conformations (Figure 1b). This is an indirect way to take into account target flexibility in docking and VS through pre-generated discrete conformations (ensemble-based VS). One of the earliest implementations is the “relaxed complex scheme” developed by McCammon and co-workers [43,44]. In this framework, an extensive MD simulation of the protein in the apo form is performed. Then, snapshots are extracted from the trajectory at regular intervals, and each of them is targeted in the subsequent docking exercise. This approach has been extensively used in the literature, and several variants and/or extensions have been reported [45]. However, in the context of VS, a major problem is to appropriately choose the protein structures used to build the ensemble. Indeed, it has been shown that an excessively large number of protein conformations may worsen rather than improve VS performances [27]. Cluster analysis approaches can therefore be helpful in reducing redundancy in the conformation set and in ensuring that only significantly dissimilar structures are included. Even in this case, enhanced sampling methods can be used to further increase the variability of the protein conformations [46].

### 3.2. Fully Dynamic Solvent Mapping

Fragment-based drug design (FBDD) is another SBDD technique, which complements docking and VS approaches in the search for new lead candidates. While docking and structure-based VS are concerned with identifying the binding mode for full-size molecules, FBDD deals with small fragments. Once the binding mode of such fragments is determined, a linking procedure is followed in order to join the building blocks and reconstruct a lead-like molecule. This de novo design procedure has several advantages over conventional strategies. It allows the exploration of novel chemical space, leading to increased ligand efficiency. It also ensures a better tuning of the design process, hopefully leading to higher hit rates [47]. From a computational standpoint, FBDD can also, in principle, be addressed through molecular docking. However, notwithstanding several successful reports, it is a common opinion that fragment docking is particularly challenging, mostly because the suboptimal performance of scoring functions becomes highly detrimental for small fragments [47]. Indeed, fragments often show multiple binding modes. Moreover, because of the lower affinity for the target, the boundary between a correct and incorrect pose is much more blurred than with lead-like ligands.

**Figure 2:**
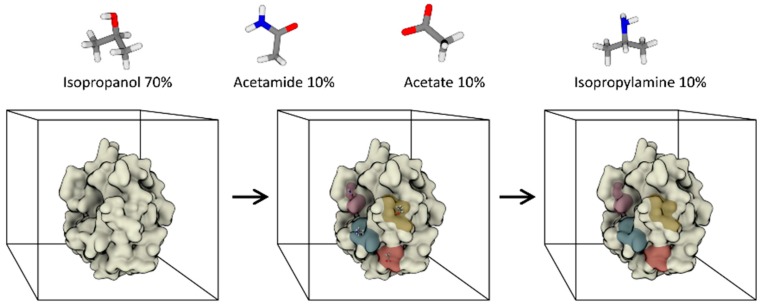
Schematic representation of hot-spots identified through solvent mapping approaches. The displayed co-solvent mixture is taken from [54].

Several approaches have been proposed to address computational de novo design, ranging from the pioneering GRID program developed by Goodford [48], through to the Multiple Copy Simultaneous Search (MCSS [49]) and related methods [50]. Carlson and co-workers recently reviewed these and other approaches [51,52], so we will not discuss them further here. Rather, without aiming to be exhaustive, we will focus on solvent mapping methods, which represent the first application of fully dynamic frameworks to the prediction of protein-ligand interactions (hot-spots). In analogy to the multiple solvent crystal structure (MSCS) method [53], where crystal structures are solved in the presence of multiple organic solvents, solvent mapping algorithms attempt to map the propensity of selected functional groups (probes) to bind the surface of a given target. To do so, simulations are performed with binary or ternary solvent mixtures, and the hot-spots for each probe are identified by measuring the occupancy of volume elements in a Cartesian grid encompassing the entire simulation box (Figure 2).

Notably, the co-solvent/water competition is explicitly taken into account, thus providing better estimates of the energetics of binding with respect to simpler scoring functions. The first co-solvent simulation framework was developed by Barril and co-workers in 2009 [55]. In this first implementation, nowadays known as MDmix, the authors used an isopropanol/water binary mixture that corresponded approximately to a 20% volume/volume concentration. The approach outperformed molecular docking by naturally including full solute and solvent flexibility. It also allowed the binding free energy of the probes to be mapped as a continuous scalar function by computing the probability ratio between volume elements in the 3D space. More recently, the framework has been extended to other mixtures [56], and an accurate evaluation of the role of target flexibility has been addressed [57]. In contrast to MDmix, the so-called Site-Identification by Ligand Competitive Saturation (SILCS) framework by Guvench and MacKerell originally used a propane/benzene/water ternary mixture [58]. The rationale for this choice of co-solvents was to take into account hydrophobic (propane) and aromatic (benzene) probes, in addition to hydrophilic ones (water). Notably, repulsive potentials were used to prevent aggregation between the highly concentrated (≈1 M) hydrophobic co-solvents. Several other solvents were then taken into account in later implementations [59], as well as advanced simulative techniques (grand canonical-Monte Carlo MD) [60]. The MixMD method by Carlson and co-workers focuses instead on the use of binary mixtures of water-miscible organic solvents, thus avoiding the use of artificial repulsive terms [61]. Meanwhile, Bakan et al. devised what is perhaps the most rigorous energetic analysis of probe hot-spots reported to date [54]. Notably, the solvent mapping framework is robust enough to address open issues other than binding-site detection. For example, MixMD has been used to identify allosteric pockets [62], while cryptic site detection was recently investigated by Favia and co-workers [63]. Finally, coarse-grained variants can also be designed. One such application comes from Ferraro et al., who mapped cholesterol binding sites on the surface of a homology-derived model of the serotonin transporter [64].

Because of the enormous potential of solvent mapping, and its intrinsically dynamic nature, we consider this technique to be a milestone in computational drug discovery. Even though it cannot be considered a direct precursor of dynamic docking methods, it nonetheless demonstrated the benefits of MD-based methods compared to static and simplified descriptions of binding.

## 4. Dynamic Docking

The approaches described in the previous Section involve MD simulations either directly (such as in solvent mapping) or indirectly (docking refinement and/or re-scoring). Dynamic docking simulations are distinguished from these approaches by the possibility of characterizing the protein-ligand binding process at a fully dynamic level. From a computational perspective, dynamic docking fully exploits the advances in sampling strategies and an unprecedented computational power. However, in analogy to static docking methods, there are two conceptual layers of complexity. The first is related to the ability to generate binding modes, which strongly depends on the sampling strategy used (“biased” or “unbiased”). The second layer concerns the evaluation of the reliability of the identified “poses”. This can be achieved by estimating the free energy of binding or by relying on statistical arguments.

### 4.1. Sampling Strategies

#### 4.1.1. Biased MD Approaches

In recent years, improvements in hardware have allowed MD simulations to capture in detail the full protein-ligand binding process (see below). However, many research groups started to explore these events long before the required computational resources were actually available. This was made possible by the development of smarter sampling strategies, which are nowadays known as “enhanced sampling methods”. These methods usually fall into two general categories: those that rely on *collective variable* (CV) *biasing*, including umbrella sampling [65], steered MD [66], and metadynamics [67], and those based on *tempering*, including replica exchange MD (REMD [68]), accelerated MD (aMD [69]), potential scaled MD [70], and multicanonical MD (McMD [71]). The main advantage of using the first type of method is that the sampling is enhanced toward the specific event of interest by biasing the MD simulations along chosen CVs, which are functions of the atomic coordinates. In this way, free-energy barriers can be efficiently crossed. However, it can be difficult to choose the right set of CVs a priori, particularly when the system’s reaction mechanism is not yet known. In the latter case, *tempering* methods seem to be more appropriate since they act by heating all degrees of freedom of the system or by modifying the Hamiltonian [3]. Below, we highlight key examples where enhanced sampling methods of both categories have been applied in the context of dynamic docking.

##### CV Biasing Methods

**Figure 3:**
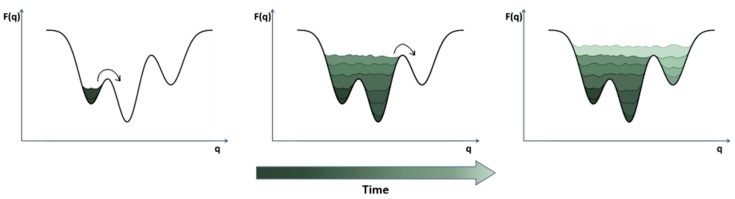
In metadynamics the bias is applied to a CV (*q*) in order to fill the underlying free energy (*F*(*q*)) and discouraging the system to visit already sampled states.

One of the earliest enhanced sampling methods used to characterize the ligand binding process is the metadynamics method introduced by Laio and Parrinello in 2002 [67]. In metadynamics, a history-dependent repulsive potential acting on a few CVs is added to the underlying dynamics, discouraging the system from exploring previously visited regions of the CV space (Figure 3). In 2005, Gervasio et al. conducted the first application of metadynamics to ligand binding. To the best of our knowledge, this is the first example of dynamic docking. The authors used metadynamics to successfully reproduce the docking geometry and the experimental binding free energy for four complexes: β-trypsin/chlorobenzamidine, immunoglobulin McPC-603/phosphocoline, and cyclin- dependent kinase 2 (CDK2)/staurosporine [72]. A few years later, Provasi et al. reported another successful example of the utility of metadynamics for simulating protein-ligand binding events. Here, they investigated the binding pathway of the nonselective antagonist naloxone to the alkaloid binding pocket of a delta opioid receptor [73]. Notably, the authors accurately assessed the association constant from the free-energy profile reconstructed through metadynamics. This was made possible by efficiently sampling the ligand in the bulk region, and allowing multiple binding/unbinding events, which are the key to accurately determining the binding free energy. To do so, they confined the unbound state of the ligand through the use of a conical shaped restraint whose contribution to the association constant can be taken into account analytically. Similarly, in 2013, Limongelli et al. developed the so-called funnel metadynamics, in which the previously described confinement is replaced by a funnel-shaped restraint, further reducing the space to explore for the ligand in the bulk [74]. Despite the successful results in predicting the mechanism of ligand binding, metadyamics suffers the same drawbacks of any other CV-based method, i.e., the need to choose an optimal set of CVs [75]. Moreover, the simulation time increases exponentially with the number of CVs, and the metadynamics performance rapidly deteriorates. This makes it difficult to accurately simulate systems characterized by a high degree of complexity. In 2007, to overcome these difficulties, Laio et al. developed a new method, bias-exchange metadynamics, which combines concepts of metadynamics and replica exchange (described below) [76]. In particular, multiple metadynamics simulations are performed in parallel and exchanged at fixed time intervals. Each replica is biased with a time-dependent potential acting on a different CV, thus alleviating the problem of CV selection. In 2009, Pietrucci et al. used the bias-exchange metadynamics technique to successfully describe the binding mechanism of a small peptide to the HIV-1 protease [77]. Even though the authors accurately computed the free energy associated with ligand binding and unbinding as a function of 7 CVs, almost 2 μs of simulation were required to converge the free energy. Despite this, they managed to characterize the kinetics of the binding/unbinding process using a discrete-states kinetic model, including the relevant metastable states along the recognition pathway. Another variant of metadynamics, reconnaissance metadynamics [78], provides a valid alternative to bias-exchange in considering a larger set of CVs. In particular, reconnaissance metadynamics is a machine-learning approach where the algorithm tunes the applied bias using data obtained from short MD simulations. Compared to conventional metadynamics, this procedure relieves the user of the a priori selection of a small number of CVs, and thus provides a way to efficiently explore previously uncharacterized mechanisms. In 2012, Soderhjelm et al. applied reconnaissance metadynamics to identify and score protein-ligand binding poses of the well-known trypsin-benzamidine system [78].

##### Tempering Methods

**Figure 4:**
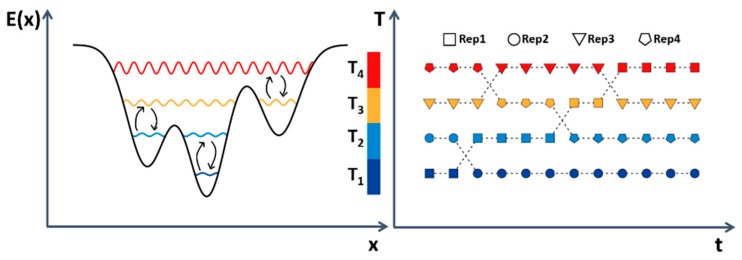
REMD does not rely on *a priori* definition of CVs. Several replicas of the system at different temperature (*T*) are simulated independently with the possibility to exchange coordinates at regular intervals.

Of the various tempering methods, the REMD method has emerged as one of the most widely used techniques to enhance conformational sampling [79]. In classical REMD, several replicas of the system are simulated independently in parallel, at different temperatures. At regular intervals, exchanges between neighboring pairs of replicas are attempted according to a Metropolis acceptance criterion (Figure 4). Because an efficient exchange requires a significant overlap of the potential energies sampled at adjacent temperatures, a high number of replicas is typically required for the method to be effective. A valid alternative to temperature REMD is provided by Hamiltonian REMD (H-REMD), in which the different replicas are simulated at the same temperature while the system’s force field is modified. An advantage of H-REMD compared to classical REMD is the possibility of varying only part of the Hamiltonian of the system among the replicas, improving the exchange probabilities [80]. This computational framework was adopted by Luitz et al. to obtain the correct binding modes for protein-ligand systems through explicit solvent simulations [81]. In particular, the H-REMD approach was based on softening the ligand-protein non-bonded interactions along the replicas in order to prevent the sampling of irrelevant states before reaching the native binding mode. The method was tested on three different systems: human FKBP protein (FKBP-52) in complex with the high-affinity ligand FK506 and with the lower affinity ligand SB3, peptide-binding domain of murine MHC class 1 molecule in complex with a viral antigen. aMD is another enhanced sampling technique that does not rely on the a priori definition of CVs. aMD speeds up the configurational space sampling by locally adding a non-negative boost potential to the system’s potential energy. The potential energy is added only to those regions of the potential energy that are below a certain threshold energy value, while leaving those above this level unaltered [69]. Recently, Kappel et al. used aMD simulations to simulate processes of ligand binding to the M3 muscarinic receptor, a G-protein-coupled receptor (GPCR) [82]. In particular, this work used long-timescale aMD simulations (hundred-nanosecond timescale) to identify the metastable ligand-binding sites of three known molecules: the antagonist tiotropium, the partial agonist arecoline, and the full agonist acetylcholine (Ach). Reweighting procedures recover the canonical distribution, from which the free-energy landscape can be calculated. However, it is still challenging to estimate the exact population for each configuration. This is because the reweighting procedure is subject to a statistical error, especially when longer timescales are simulated. In this work, where aMD simulations were performed on the 100–1000 ns timescale, the authors focused on identifying metastable ligand-binding sites on the M3 receptor, in agreement with unbiased MD simulations, in a significantly shorter time (about 80 times faster for Ach). In 2007, Kamiya et al. provided interesting results by performing McMD simulations to successfully dock the inhibitor tri-*N*-acetyl-d-glucosamine [83]. McMD is an enhanced sampling method, in which a random walk sampling through the energy space is made possible by the bias applied to the system [71]. In the McMD method, higher energy states and lower energy states have an equal probability of being sampled because different temperature regions defined by the bias are simulated simultaneously. A merit of the McMD method is that the canonical ensemble can be reconstructed at 300 K relatively easily by a reweighting procedure. Recently, Bekker et al. also performed long McMD simulations to dock the inhibitor CS3 to cyclin-dependent kinase 2 [84]. To accelerate the reproduction of the native complex, the ligand was restrained in a cylindrical region near the binding pocket. In addition, after having identified the correct binding mode, they accurately predicted the binding free energy by TI in accordance with the experimental data.

#### 4.1.2. Unbiased MD Approaches

Improvements in computer architectures (i.e., GPUs, specialized hardware such as Anton, distributed computing networks such as GPUGRID.net) have allowed several groups to reach and break the millisecond barrier in MD simulation timescales. Ten years ago, the research community hoped to one day simulate a spontaneous and unrestricted drug-binding event without applying any bias to the system. This is now possible. We stress that we consider “unbiased approaches” to include simulative setups and protocols that do not alter the dynamics through external forces. This does not necessarily mean that the dynamics is always continuous. As we will see later, some workarounds to reduce the computational burden do indeed rely on discontinuous trajectories.

##### Brute-Force MD

The earliest attempt of using unbiased MD to reconstruct a binding event was made by Wang and Tajkhorshid in 2008 [85]. Thanks to the presence of a strong electrostatic potential in the binding site of the target, the mitochondrial ADP/ATP carrier, it has been possible to record the binding event of an ADP molecule initially positioned in close proximity of the receptor to a deeply buried site. Their seminal work paved the way to all the following efforts aimed at studying a complete and spontaneous drug binding event through MD (see Table 2). The first example of unbiased dynamic docking was reported by Shan et al. in 2011 [86]. The authors randomly placed two Src kinase inhibitors within a simulation box together with their target protein and let them freely diffuse to their binding site. The authors recorded several spontaneous binding events forming complexes nearly identical to those resolved by X-ray crystallography. They also identified a previously unknown allosteric pocket, highlighting the potential of these simulations as tools for standard drug discovery programs. Buch and co-workers followed a similar path to completely reconstruct the benzamidine to trypsin binding process in terms of pathway and related energetics. Their work produced insights into the mechanism of association of a drug to its target without neglecting intermediate states [87]. Similar works were then conducted for membrane receptors (the *β*-adrenergic GPCRs coupled to agonist and antagonist small molecules [88] and the spontaneous binding of tiotropium and acetylcholine to M2/M3 muscarinic receptors [89]) and, recently, for the purine nucleoside phosphorylase enzyme and its pM inhibitor, the transition state analog DADMe-immucillin-H [90]. Similarly, researchers have made it feasible to use unbiased MD simulations for fragment-to-lead development [91]. Here, in addition to reproducing all the crystallographic poses of carboxythiophene fragments present in the X-ray structure of AmpC *β*-lactamase, the trajectory analysis discriminated between distinct binding modes from both a thermodynamic and a kinetic standpoint [92]. Hence, this dynamical approach to docking shed light on the ligand’s route to binding, helping to characterize the major energetic barriers along this path and the factors that may influence them. These factors include transient interactions, dehydration, ligand geometry, and so on, many of which are mistreated by traditional docking techniques. Dynamic docking can also be used to reveal binding sites and poses of known binders even when there is no previous knowledge of them. Dror and co-workers studied the M2 muscarinic acetylcholine receptor along with a number of experimentally identified allosteric modulators for which no crystal structure was available. They identified an extracellular-facing vestibule, to which several modulators can bind, observing that the binding driving force was a set of cation-π interactions rather than the previously proposed aromatic-aromatic contacts. Notably, their results were validated by mutagenesis analysis and radioligand binding experiments, confirming the predictive power of these simulations [93].

In sum, brute force unbiased MD simulations were a striking milestone in the development of dynamic docking. However, they also highlighted their main limitations. Indeed, the binding of a small molecule to its binding pocket can still be considered a rare event, especially when it comes to ligands with unfavorable on-rates (i.e., low *k*_on_). To this end, the easiest and most popular approach to overcoming this hurdle was to place more than one ligand molecule in the simulation box, increasing the probability of observing a binding event. This strategy may be of some help. However, multiple long trajectories are still needed to thoroughly sample the configurational space and collect enough statistics. Therefore, it is not surprising that most of the above-reported applications took advantage of specialized hardware or massive computing architectures to carry out the simulations. To this end, in the context of unbiased simulations, several solutions have been proposed to make the use of dynamic docking more practical and effective (see Table 2 for a comparative list). These strategies include adaptive sampling methods, supervised approaches, and multiscale modeling, and are described in the following subsection.

##### Discontinuous Approaches

Among discontinuous approaches, adaptive sampling are a class of unbiased MD methods relying on the Markov State Model (MSM) framework [94,95]. They aim to identify undersampled states during the binding process in order to run new simulations from these points (a process usually termed “seeding”) so as to lower the statistical error related to thermodynamic quantities. In this way, a few ultra-long simulations are replaced by many short ones, saving valuable computational time. In the earliest implementations, human intervention was essential to manually select the simulation restarting point [96]. In 2014, however, Doerr and De Fabritiis, presented an automated protocol that reconstructed the binding process for the trypsin-benzamidine system in a totally unsupervised fashion [97]. The automated learning method achieved a converged binding affinity one order of magnitude faster than classical sampling. They implemented their strategy in a freely available environment (known as HTMD [98], High Throughput MD, which is also suitable for replacing MSM with other adaptive sampling algorithms). They thus elucidated the cooperative recognition mechanism of ionic cofactors and substrate in the myo-inositol monophosphatate enzyme [99], and the binding process of the lipid inhibitor ML056 to the sphingosine-1-phosphate receptor [100]. The latter is an informative example because it describes the diffusion of the lipophilic ligand ML056 from the bulk solvent to the membrane bilayer, prior to moving to its orthosteric binding site. Unlike other transmembrane receptors, this binding site is not accessible from the extracellular environment.

A strikingly different approach is the supervised molecular dynamics (SuMD) protocol devised by Sabbadin and Moro [101]. In adaptive sampling methods, seeding is used to resample under-sampled states. In the SuMD implementation, however, simulations that are unlikely to lead to a productive binding event are discarded on the fly by a tabu-like algorithm. During short simulation windows, at defined time intervals, the distance between the center of masses of the ligand and the binding site is saved and subsequently fitted in a linear function. If the slope of the resulting line is negative, it means that the ligand is moving toward its binding site, and the simulation is evolved. Otherwise, the current simulation is interrupted, and a new run is restarted from the last saved coordinates through velocity reassignment. Finally, at a user-defined distance value, the supervision algorithm is switched off and continuous dynamics is restored. The method has been tested against a number of complexes of both globular and membrane proteins [102,103] and was effective in reproducing ligand-receptor complexes in nanosecond-range timescales. On the other hand, its limitation is the need for *a priori* knowledge of the binding site location. In principle, at least, this precludes the possibility of discovering novel and unexplored binding sites as in brute force dynamic docking simulations. Moreover, while the protocol has proven valuable in identifying relevant metastable states during the recognition process, because of the discontinuous nature of the trajectories, the binding route followed by the ligand is not necessarily close to the minimum free-energy pathway.

Similarly, multiscale approaches aim to reduce the time that a ligand spends diffusing in the solvent probing its binding site, which is not a relevant part of the association pathway. The idea is to limit the full atomistic resolution and computationally expensive MD simulation to those regions that are close to the binding site. Very recently, Zeller et al. introduced a multiscale approach to dynamic docking that allows the binding kinetics to be evaluated. As a test case, they used two H1N1 neuroaminidase inhibitors, oseltamivir and zanamivir [104]. Their implementation used Brownian Dynamics (BD) [105] when the distance between the ligand and binding site was more than a properly defined value. In this region, ligands and protein are treated as rigid bodies that undergo translational and rotational diffusion in an implicit continuum solvent. When the ligand reaches the so-called encounter surface, BD switches to all-atom MD and the solvent is treated explicitly. They succeeded in reproducing X-ray crystal structures. However, as they stated, this approach would be useful only in cases where binding pathways are diffusion-controlled. The usual simulation limitations apply when the binding involves large conformational changes. A similar MD/BD approach was proposed few years earlier by Amaro and co-workers [106]. In such an approach, the atomistic dynamics relies on milestoning, and the methodology is mostly suited to describe (un)binding kinetics and to compute the corresponding rates rather than reproducing drug-target complexes. Recently, a fully automation of the protocol led to the release of the so-called SEEKR software package, which is intended to provide a user friendly interface to assist setup and analysis of simulations [107].

### 4.2. Estimation of Experimentally Accessible Observables

As with static molecular docking, sampling the experimental binding mode is necessary but not sufficient for the exploitation of dynamic docking in prospective drug discovery. Indeed, proper strategies are required to discriminate between stable binding modes and “false positives” (binding modes differing from the experimental one). The false positive concept is more complex than in conventional docking. Here, it includes both “wrong” binding modes, due to inaccuracies in the potential energy function and/or sampling limitations, and true metastable states identified along the recognition pathway, which may be required to reach the final bound state. Clearly, the latter adds value to dynamic docking, and should be distinguished from the former situation. It follows that an accurate prediction of binding free energy and kinetics is of crucial importance to evaluate which binding mode has to be taken into account for predictive studies.

By exploiting enhanced sampling methods, biased MD approaches to dynamic docking are naturally endowed with their own free-energy estimators. We will not discuss the advantages and drawbacks of each approach here because the relevant details can be found elsewhere [8]. Concerning unbiased dynamic docking, the most straightforward way to assess the free-energy difference associated with any process is to evaluate the probability ratio (*ρ*) between the initial and final states. In the context of dynamic docking, where two separated molecular entities bind to form a single complex, a correction term must be added, leading to [108,109]:

(6) $$\Delta G_{b}^{{}^{\circ}}=-k_{B}T\ln\left(\frac{\rho_{\mathrm{bound}}}{\rho_{\mathrm{unbound}}}\right)-k_{B}T\ln\left(\frac{V^{\prime }}{V^{0}}\right)$$

**Figure 5:**
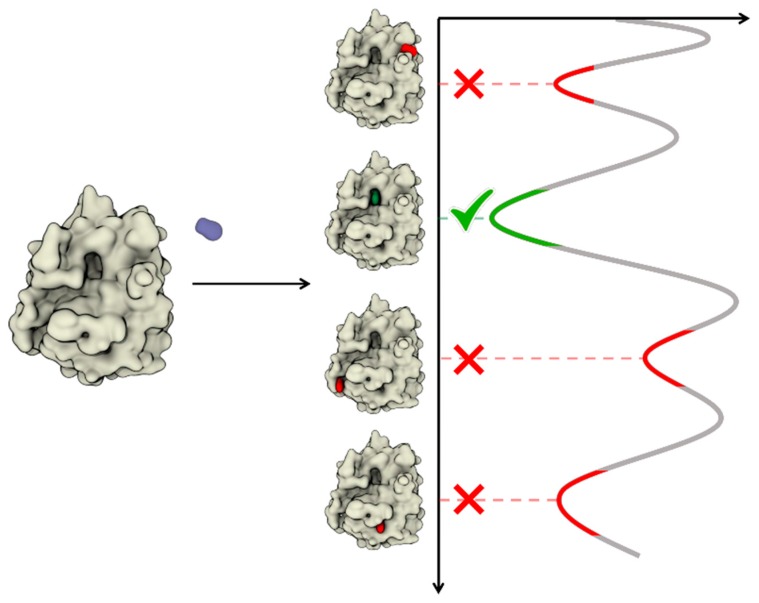
Schematic representation of unbiased MD approaches to dynamic docking. The most populated state should, in principle, correspond to the energetic minimum.

The second term on the right-hand side of Equation (6) accounts for the fact that the reference volume (*V*′) in the simulations is different from the experimental volume (standard volume, *V*^0^ i.e., 1661 Å^3^). It ensures a direct comparison between computational and experimental free energies (standard free energy of binding). An equivalent strategy is followed when solvent mapping is used to provide quantitative estimates [54,57]. Nevertheless, evaluating the free energy as a probability ratio is extremely difficult since many transitions are required to achieve proper statistical confidence. This is even more problematic in the context of brute force dynamic docking, where it is already challenging to observe a single docking event. As a practical workaround, rather than an energetic comparison of distinct binding modes, unbiased dynamic docking often relies on the statistical analysis of the obtained trajectories. Thus, the most populated binding mode is reasonably assumed to be the energetically preferred mode from among the ensemble of identified stable states (see Figure 5 and Table 2). A remarkable exception comes from the HTMD framework developed by De Fabritiis and co-workers [98]. Here, by exploiting MSM and adaptive sampling, the binding pose is indeed identified by converging the binding free energy evaluated through Equation (6).

Another obvious advantage of dynamic docking over conventional approaches is the possibility of characterizing the mechanistic steps of the entire protein-ligand binding process. This, in principle, allows the disclosure of potential metastable states or even allosteric pockets. It is highly relevant for drug discovery. Moreover, provided that adequate sampling is achieved, kinetic observables such as the association and dissociation constant (*k*_on_ and *k*_off_, respectively) can also be determined (mostly by relying on MSM approaches). This is a crucial aspect of unbiased dynamic docking because it expands the predictive power of computational methods applied to SBDD. Notably, the estimation of kinetic constants is the focus of the most sophisticated approaches developed so far [90,97,98,104,107]. In particular, from a drug discovery standpoint, accurately predicting the *k*_off_ can be as important as estimating the binding free energy. This is because the dissociation of protein-ligand complexes, which follows an exponential decay with the characteristic time *τ*_R_ = 1/*k*_off_ (residence time), is a better indicator of in vivo drug efficacy than the equilibrium constant [5]. Therefore, optimizing the residence time via rational design is a breakthrough for computational drug discovery. It is nonetheless true that computing the residence time through unbiased approaches is still a daunting problem. This is due to the long timescales involved in the dissociation of protein-ligand complexes, which can last from milliseconds to minutes (or more). The increasing computational power will certainly alleviate this aspect. In addition, several workarounds have been proposed to accelerate the calculations. These mostly rely on enhanced sampling methods and were recently reviewed elsewhere [110,111].

### 4.3. Current Challenges and Future Directions

In spite of the obvious advantages provided by dynamic docking over simplified and static descriptions, the methodology is not free from challenges and indirect limitations. First and foremost, dynamic docking is a rather novel concept in drug design, and more validation is certainly required to better discern the relative advantages and drawbacks among the several variants and approaches. Indeed, to the best of our knowledge, the only prospective validation was provided by Dror and co-workers in the context of brute force MD to study ligand binding to the M2 muscarinic acetylcholine receptor [93]. It is foreseeable that, as soon as more powerful computational architectures will be available, more validation studies will be performed on diversified test cases.

As already mentioned, the computational time required to accomplish dynamic docking simulations probably remains the greatest challenge of the methodology. This aspect is particularly relevant for industry, where speed is a mandatory requirement for any drug design program. It also explains the reason why a number of methods have been developed to reduce as much as possible the time needed to observe a ligand binding event or to estimate relevant observables within a given accuracy. In this context, biased-MD approaches, which can be considered to some extent “old-fashioned”, are still appealing as they provide a computationally cheaper way to dynamic docking. Again, proper prospective validations will be helpful in determining whether and to what extent it is worth to replace static molecular docking with a fully dynamic description.

Another often overlooked difficulty is related to the generation of huge amount of data. So far, dynamic docking has been mostly employed to study a limited number of protein-ligand complexes. However, in the context of a paradigm shift, dynamic docking is expected to replace docking and VS. Therefore, the disk space required to store a large number of trajectories will most certainly become a technological challenge as serious as the computational power needed to generate the data. From a different standpoint, dynamic docking intrinsically deals with big data, and several machine learning approaches are already available to make optimal use of trajectories (e.g., adaptive sampling).

Finally, we wish to mention that, so far, a full integration of dynamic docking into professional and user friendly packages is still lacking. In principle, brute force dynamic docking can be performed with any software equipped with an MD engine, whereas in-house modifications of the original codes and/or external plugins are required by discontinuous approaches. Concerning biased dynamic docking, most of the widely used MD software nowadays come with their own biased MD support. Besides, the PLUMED software [112] has played a major role in providing the most flexible plugin to enhanced sampling and, therefore, to biased dynamic docking. These aspects are summarized in Table 3, and reveal a further difference between dynamic and static molecular docking. Indeed, while several software packages for static molecular docking have been available since the beginning (Table 1), a different situation is portrayed for dynamic docking. In this case, several programs (and combination thereof) can be exploited, but no dedicated platforms are currently reported. This also implies that experienced MD users are needed to perform and/or analyze dynamic docking. From this standpoint, a substantial contribution towards fully automated and user friendly protocols is provided by the BiKi Life Sciences [113], HTMD, and SEEKR software interfaces. It is likely that these and other software packages will ultimately lead to the paradigm shift from molecular mechanics to statistical mechanics triggered by dynamic docking.

## 5. Conclusions and Perspectives

Since its debut in the field of molecular discovery, docking simulations have proven to be invaluable tools for discovering new compounds of pharmaceutical interest. However, to make sense of often-discordant experimental results, a dynamical approach is mandatory to enhance the hit-to-lead process.

The studies reviewed herein demonstrate how a move to dynamic docking could offer great potential and utility in understanding how ligand and proteins cooperatively exert their mechanism of action and in characterizing binding pathways and related observables. Indeed, due to intrinsic limitations in treating protein flexibility and explicit solvation effects, traditional approaches have mostly underestimated the relevance of accurately estimating the binding free energies and kinetic constants. Because the timescales of biological events exceed those that can be commonly simulated, the earliest biased MD approaches were the only instrument for gaining insights into mechanisms and energetics. Nevertheless, we believe that analysis of the data generated by unbiased MD approaches and access to the necessary computational power will be made feasible by advances in hardware architectures and machine-learning techniques. This will transform dynamic docking into an everyday technique for drug discovery programs.

## Figures and Tables

**Table 1:** Examples of some of the most popular currently available docking software. For a comprehensive list see [15].

Software	Searching Algorithm	Native Scoring Function ^1^	License
AutoDock [16]	Stochastic	Force-Field based	Free for Academia
DOCK [17]	Systematic	Force-Field based	Free for Academia
FlexX [18]	Systematic	Empirical	Paid
Glide [19]	Systematic	Empirical	Paid
GOLD [20]	Stochastic	Force-Field based	Paid
ICM [21]	Stochastic	Force-Field based	Paid
MOE [22]	Stochastic	Force-Field based	Paid

^1^ Usually, the user can choose among several, often customizable, scoring functions. Here we report the type of scoring functions originally developed with the docking program.

**Table 2:** Comparative time scales of brute force MD versus discontinuous approaches as reported in retrospective studies. Owing to the inherent difficulties in comparing timescales of several short trajectories, adaptive sampling is not considered in this table.

Author (Year)	Complex	Multiple Ligands	No. of Runs	Aggregate Time	Productive Runs ^1^	Time to Binding
**Brute Force MD**						
Shan et al. (2011)	PP1/Src kinase	y	7	115 µs	3	15.1–1.9–0.6 µs
	Dasatinib/Src kinase	y	4	35 µs	1	2.3 µs
Buch et al. (2011)	Benzamidine/Trypsine	n	495	49.5 µs	187	15–90 ns
Dror et al. (2011)	Dihydroalprenolol/*β*_2_AR	y	40	111.8 µs	5	NA
	Alprenolol/*β*_2_AR	y	10	14 µs	1	NA
	Propranolol/*β*_2_AR	y	21	35.7 µs	0	-
	Isoprotenerol/*β*_2_AR	y	1	15.0 µs	0	-
	Dihydroalprenolol/*β*_1_AR	y	10	55.5 µs	2	NA
Kruse et al. (2012)	ACh/M3 R	y	1	25 µs	1	9.5 µs
	Tiotropium/M3 R	y	3	18 µs	0	-
	Tiotropium/M2 R	y	3	16.2 µs	0	-
Decherchi et al. (2015)	DADMe-immucilin-H/PNP	y	14	7 µs	3	340 ns
**Discontinuous Approaches**						
Sabbadin et al. (2014)	ZM241385/hA_2A_	n	3	-	1	59 ns
	T4G/hA_2A_	n	3	-	1	62 ns
	T4E/hA_2A_	n	3	-	1	105 ns
	Caffeine/hA_2A_	n	3	-	1	15.2 ns
Cuzzolin et al. (2016)	Ellagic Acid/CK2	n	3	-	0	-
	SAPS/GSTP1-1	n	3	-	2	27–19 ns
	Benzen-1,2-diol/PRDX5	n	3	-	3	17.4–31.2–18 ns
	(*S*)-naproxen/HSA	n	3	-	0	-
	(*S*)-fluoxetin/LeuT	n	3	-	0	-
	NECA/hA_2A_	n	3	-	0	-
Zeller et al. (2017)	Oseltamivir/neuraminidase	n	676	50.0 µs	~20	NA
	Zanamivir/neuraminidase	n	606	35.7 µs	~20	NA

^1^ “Productive” refers to simulations that reproduced the crystallographic pose within a given RMSD threshold.

**Table 3:** Examples of MD software that can be used to perform dynamic docking simulation.

Software	GPU Support	Biased MD Support	PLUMED 2.3 Patch Available	License
MD Engines				
ACEMD [114]	x	x	x ^1^	Free Serial Version (for Academia)
AMBER [115]	x	x	x	Paid
CHARMM [116]	x	x		Free Serial Version
Desmond [117]	x	x		Free for Academia
DL_POLY [118]	x	x	x ^1^	Free for Academia
GROMACS [119]	x	x	x	Free
LAMMPS [120]	x	x	x	Free
NAMD [121]	x	x	x	Free
ORAC [122]		x		Free
Tinker [123]	x			Free
Software Interfaces				
BiKi Life Sciences	-	-	-	Paid
HTMD	-	-	-	Free Basic Version (for Academia)
SEEKR	-	-	-	Free

^1^ PLUMED is natively supported by the MD engine.
